# Two cases of probable Neuro-Behçet syndrome treated with autologous HSCT

**DOI:** 10.3389/fimmu.2026.1864802

**Published:** 2026-07-13

**Authors:** Charlotte Schubert, Lea I. Walter, Marina Herwerth, Imke Metz, Jakob Nilsson, Ilijas Jelcic, Patrick Roth, Nicolaus Kröger, Ina Kötter, Christoph Heesen, Vivien Häußler, Veronika Kana

**Affiliations:** 1 Institute of Neuroimmunology and Multiple Sclerosis (INIMS) and Department of Neurology, University Medical Centre Hamburg-Eppendorf, Hamburg, Germany; 2 Department of Neurology, University Hospital Zurich and University of Zurich, Zurich, Switzerland; 3 Neuroscience Centre Zurich, University of Zurich and ETH Zurich, Zurich, Switzerland; 4 Institute of Pharmacology and Toxicology, University of Zurich, Zurich, Switzerland; 5 Institute of Neuropathology, University Medical Centre Göttingen, Göttingen, Germany; 6 Department of Immunology, University Hospital Zurich, Zurich, Switzerland; 7 Department of Stem Cell Transplantation, University Medical Centre Hamburg-Eppendorf, Hamburg, Germany; 8 Division of Rheumatology and Systemic Inflammatory Diseases, III. Department of Medicine, University Medical Centre Hamburg-Eppendorf, Hamburg, Germany

**Keywords:** aHSCT, Behçet syndrome, case report, myelitis, stem cell therapy

## Abstract

Neuro-Behçet syndrome (NBS) is a rare but detrimental neurological manifestation of the Behçet syndrome (BS) – a chronic multisystemic inflammatory disease. NBS is frequently associated with brainstem and spinal cord lesions, often resulting in substantial neurological disability. Atypical clinical presentations can pose significant challenges for diagnosis and treatment of NBS. Here, we report the clinical course, treatment strategies and responses of two cases with probable NBS. Both cases presented with longitudinally extensive transverse myelitis and brainstem lesions and experienced recurrent relapses as well as disease progression resistant to multiple immunotherapies. Autologous hematopoietic stem cell transplantation (aHSCT) was without beneficial effect on the disease course in both cases. In summary, NBS or probable NBS should be considered in patients with myelitis and brainstem involvement with limited treatment response. These cases illustrate diagnostic challenges and limitations of current diagnostic criteria, while also underscoring the heterogeneity of disease presentation. Immune reset by aHSCT failed to confer clinical benefit as a rescue therapy in these two patients. Further studies are required to optimize therapeutic strategies for NBS.

## 1 Introduction

Behçet’s syndrome (BS) is a systemic vasculitis of variable vessels (1, 2). Mechanistically, increasing evidence suggests a multifactorial pathogenesis involving genetic susceptibility and environmental triggers, such as infections and stress (1, 3). Neurologic involvement occurs in less than 10% of the cases and typically develops 5–6 years after onset of systemic manifestations with either parenchymal or vascular involvement. In Neuro-Behçet syndrome (NBS), central nervous system (CNS) involvement most commonly presents as immune-mediated meningoencephalitis, predominantly affecting brainstem, but may also involve basal ganglia, thalamus, cortex, white matter, spinal cord (longitudinal extensive transverse myelitis (LETM)), or cranial nerves (4–6). In cases with spinal cord involvement, neuromyelitis spectrum disorder (NMOSD) is a key differential diagnosis.

While diagnostic criteria of BS include established clinical criteria, including recurrent oral or genital ulcera, and ocular lesions, the diagnostic criteria of NBS are less well defined (1). International consensus recommendations published in 2014 (7) propose that patients fulfilling the International Study Group criteria for BS, present with compatible neurological manifestations supported by neuroimaging and/or cerebrospinal fluid (CSF) findings, and lack a more plausible alternative diagnosis (7).

Evidence guiding treatment of NBS is limited with only one small phase II trial confirming tumor necrosis factor (TNF)-α-inhibitors superior to cyclophosphamide (8). Besides TNF-α-inhibitors immunosuppressive agents such as azathioprine, mycophenolate mofetil, interferons, tocilizumab and steroids are commonly used. Experience with escalation therapies such as autologous stem cell therapy (aHSCT) remains limited, with variable outcomes reported (9). Here, we present two cases of probable NBS highlighting the diagnostic and therapeutic challenges.

## 2 Methods

We report the clinical course, diagnostic challenges, treatment strategies including aHSCT and treatment responses of two cases of probable NBS.

### 2.1 Data visualisation

Data visualisation was performed using R (version V4.3.2) with the packages ggplot2 and tidyverse. The graphical abstract was created with Biorender.com.

## 3 Results - case description

### 3.1 Case 1

**Figure 1:**
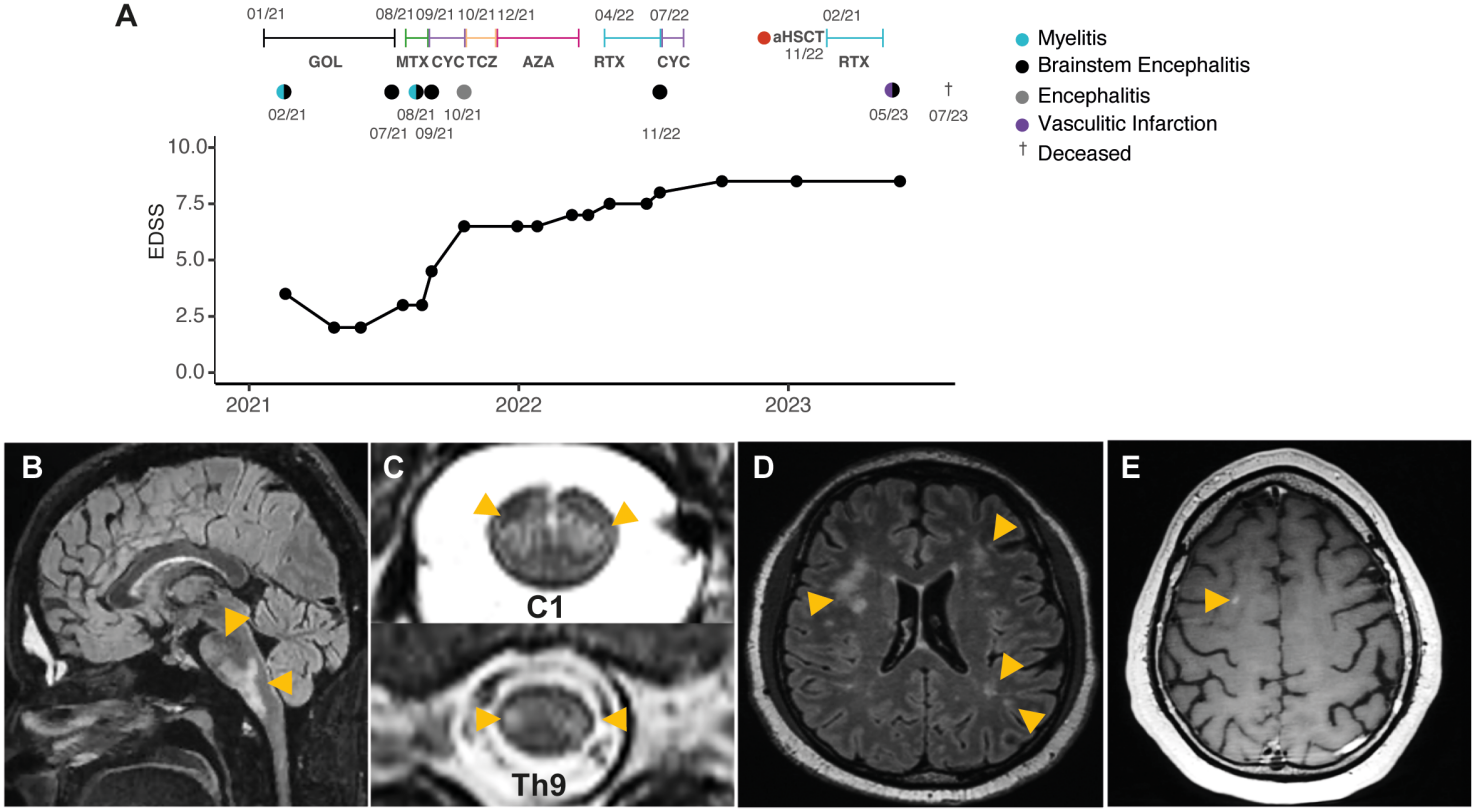
Case 1. **(A)** Disease course of patient 1 visualised by increase in EDSS = Expanded Disability Status Scale. **(B, C)** MRI before aHSCT in 02/2021 and 08/2021 with **(B)** sagittal T2 FLAIR sequence with demyelinating brainstem lesion and **(C)** axial T2-weighted sequences showing extensive T2-hyperintensive lesion from the cervicospinal junction to the thoracic cord. **(D, E)** MRI after aHSCT in 06/2023 with **(D)** axial T2 FLAIR sequence with supratentorial lesion load and **(E)** axial T1-weighted sequence with contrast enhancement. aHSCT, autologous haematopoietic stem cell transplantation; AZA, azathioprine; CYC, cyclophosphamide; GOL, golimumab; MTX, methotrexate; RTX, rituximab; TCZ, tocilizumab; ^†^death of the patient due to infection under pancytopenia.

**Figure 2:**
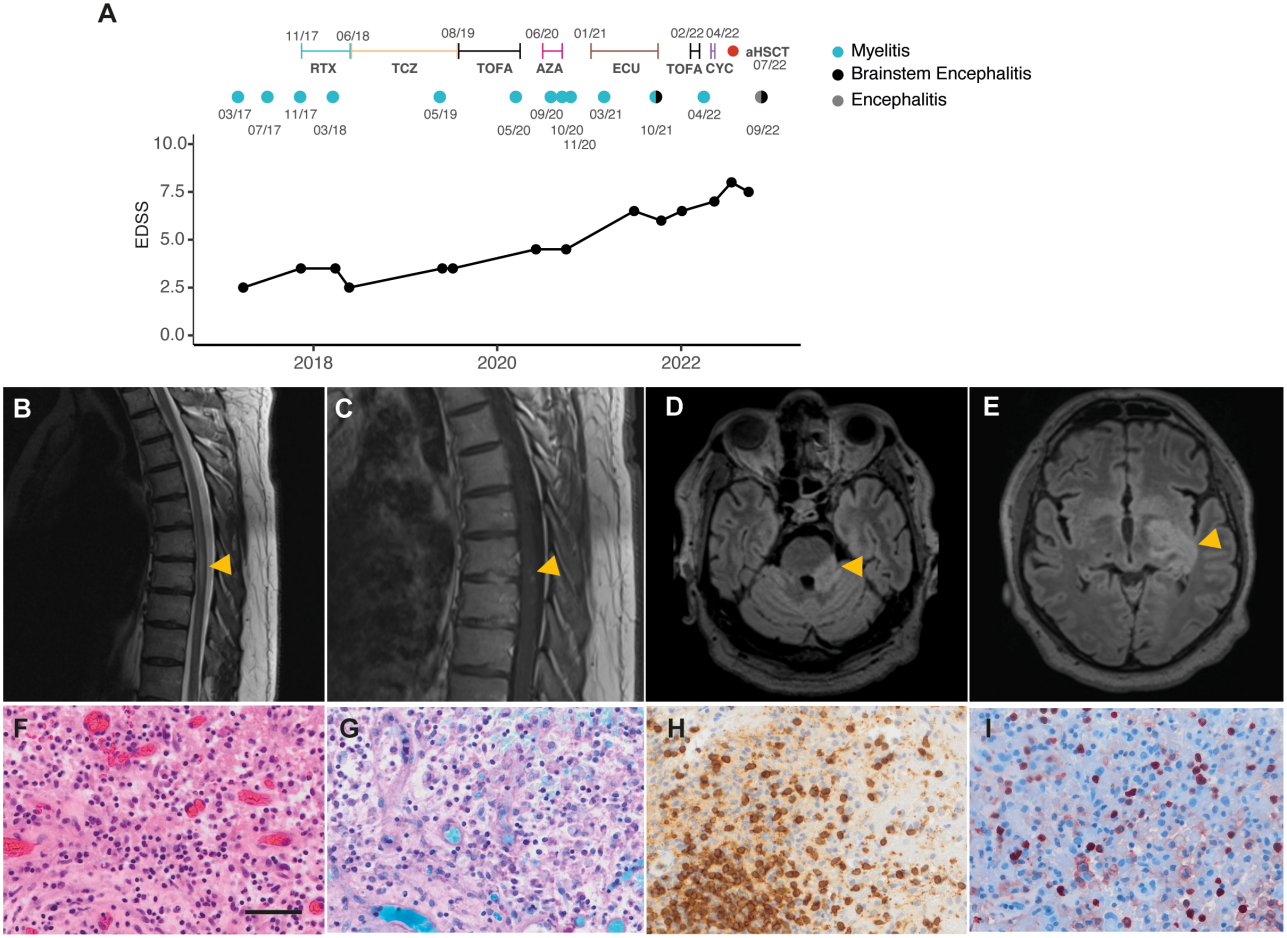
Case 2. **(A)** Disease course of patient 2 visualised by increase in EDSS = Expanded Disability Status Scale. **(B, C)** MRI before aHSCT in 10/2019 with **(B)** sagittal T2-weighted sequence of thoracic spinal cord showing longitudinally extensive T2-hyperintensive lesion from T7 to T9 and **(C)** Sagittal T1-weighted sequence of thoracic spinal cord revealing nodular gadolinium contrast. **(D, E)** MRI after aHSCT in 10/2022 with **(D)** representative image of pontocerebellar lesion as well as **(E)** insular lesion visualised in axial T2-weighted sequence. **(F–I)** Histology of inflammatory necrotic lesion consistent with Behçet disease: **(F)** H&E staining reveals necrotic and cellular lesions within the brain tissue, accompanied by an inflammatory infiltrate composed of lymphocytes, granulocytes, and numerous macrophages. **(G)** Luxol fast blue/PAS staining demonstrates loss of myelin in necrotic areas, while myelin is preserved in non-necrotic areas (not shown). **(H)** The inflammatory infiltrate contains numerous lymphocytes (CD3). **(I)** MRP14 staining highlights the granulocytic infiltrate (strongly stained polymorphonuclear cells) as well as early activated macrophages (faintly stained). Scale bar: 50 µm valid for **(F–I).** aHSCT, autologous haematopoietic stem cell transplantation; AZA, azathioprine; CYC, cyclophosphamide; ECU, eculizumab; RTX, rituximab; TOFA, tofacitinibe; TCZ, tocilizumab.

A 28-year-old patient of Kosovar descent presented in 2021 with a subacute left-sided motor hemisyndrome with hyperreflexia, dysarthria, and diplopia (**Figure 1A**). MRI demonstrated extensive hyperintense lesions in T2-weighted sequences predominantly involving brainstem and a LETM reaching from the craniocervical junction to Th10 (**Figures 2B, C**). A history of spondylarthritis under treatment with anti-TNF-α therapy (Etanercept 50mg every two weeks followed by Golimumab 50mg every three weeks) and prior oral ulcers raised suspicion for NBS. CSF showed pleocytosis (97/µl) and moderate blood-brain barrier dysfunction, without intrathecal immunoglobulin-synthesis or oligoclonal bands. Anti-aquaporin (AQP)-4-IgG and anti-myelin-oligodendrocyte-glycoprotein (MOG)-IgG (live cell-based assay) were negative. HLA-B51 was positive, while the pathergy test was negative, possibly due to prolonged corticosteroid exposure, resulting in an International Criteria for Behçet’s disease score of 3 points (10).

Despite repeated high-dose corticosteroids with transient stabilization, clinical and radiological progression over next 9 months prompted cyclophosphamide therapy. Two further severe relapses occurred within one year (**Figure 1A**), resulting in loss of ambulation, progressive dysarthria and dysphagia. Subsequent treatment with azathioprine, tocilizumab, colchicine and rituximab failed to control disease activity, resulting in severe disability (Expanded Disability Status Scale = EDSS 8.5). Almost two years after disease onset, aHSCT with cyclophosphamide and rabbit anti-thymocyte globulin (rATG) conditioning was performed as rescue therapy. Post-discharge (01/2023), the patient developed recurrent CMV-reactivation and upper gastrointestinal bleeding due to ulcerous duodenitis. Four months post-aHSCT, marginal zone lymphoma was diagnosed and treated with rituximab. At six months post-aHSCT, unilateral worsening of pre-existing arm paresis occurred. MRI revealed persistent brainstem, supratentorial (**Figure 2D**) and longitudinal spinal lesions, but also a new cerebellar inflammatory lesion and a frontal subcortical vasculitic-appearing lesion (**Figure 2E**), consistent with ongoing activity of probable NBS. The patient died from infection with pancytopenia nine months after aHSCT. In accordance with the patient’s will, no autopsy was performed.

### 3.2 Case 2

A 59-year-old male of Turkish origin presented in 2017 with a first episode of sensory myelitis (**Figure 2A**). MRI revealed a LETM from the craniocervical junction to C5 and a solitary nonspecific supratentorial lesion. CSF revealed mild pleocytosis (35/µl) with activated lymphomonocytes, granulocytes, and elevated CSF protein. There was no presence of oligoclonal bands and negative intrathecal polyspecific antiviral immune response (MRZ reaction). Anti-AQP4-IgG was negative, while anti-MOG-IgG was low positive (1:80, live cell-based assay). A second myelitic attack with motor and sensory symptoms occurred four months later. Seronegative NMOSD was suspected.

Over the next 3.5 years, the patient experienced seven myelitic attacks, predominantly LETMs (**Figures 2B, C**), and one brainstem associated attack despite treatment with rituximab, tocilizumab, tofacitinib, azathioprine, eculizumab, and cyclophosphamide, resulting in severe ambulatory impairment (EDSS 6.0; **Figure 2A**). Infliximab was not administered due to concerns regarding potential treatment induced exacerbation in immune-mediated diseases, including MS, NMOSD and MOGAD. aHSCT was performed as a rescue therapy in 07/2022 following European Society for Blood and Marrow Transplantation (EBMT) protocols for autoimmune neurological diseases such as multiple sclerosis (MS) and NMOSD, including cyclophosphamide and G-CSF mobilisation followed by intermediate intensity conditioning with cyclophosphamide and anti-T-lymphocyte-globulin to achieve immune reset (11).

Three months post-aHSCT, paraparesis worsened with new MRI lesions in pontocerebellar, temporomesial localization (**Figures 2D, E**) and in the cervicothoracic junction. CSF was without detection of bacterial, viral or fungal infection, while pleocytosis persisted containing lymphocytes and increased count of polymorphonuclear neutrophils (79%). A brain biopsy (01/2023) revealed necrotizing lesions with T-lymphocytes, granulocytes, and numerous macrophages (**Figures 2F–I**, **Supplementary Figures 1A–D**). Vessel wall inflammation was observed, but no evidence of fibrinoid necrosis or thrombosis was found (**Supplementary Figures 1E, F**). Demyelinating diseases such as MS, NMOSD, or MOG-antibody–associated disease (MOGAD) were ruled out. A loss of aquaporin-4, characteristic of NMOSD, was absent, and no pathogens were detected. Histopathology was compatible with parenchymal Neuro-Behçet disease. Classical manifestations of Behçet disease, such as oral or genital ulcers, ocular involvement, or HLA-B51 positivity, were absent. Thus, formal diagnostic criteria for BS were not met (fulfilling 2 points in the scoring system). Nevertheless, some peripheral inflammatory manifestations were observed, including intermittent elevations of C-reactive protein without infection and a history of superficial thrombophlebitis, and thus supported the diagnosis of probable NBS. At the last follow-up, the patient presented with severe spastic sensorimotor tetraparesis, sensory loss below Th8, and bladder dysfunction (EDSS 7.5), but refused further immunotherapy.

## 4 Discussion

Here we report two patients with LETM and brainstem involvement who developed a highly aggressive disease course, refractory to multiple immunosuppressive therapies and persistent disease activity despite aHSCT. Overall, the clinical presentation, lesion distribution, and treatment resistance were most consistent with probable NBD, although the patients did not fulfill established diagnostic criteria of Behçet’s disease, see also **Supplementary Table 1** (7). In patient 2 histopathological examination was decisive in supporting a diagnosis of probable parenchymal NBS (12). Given the broad range of conditions that can cause a comparable pattern of spinal cord and brainstem inflammation careful evaluation was required. The differential diagnosis of longitudinally extensive spinal cord and brainstem lesions extend beyond MS, NMOSD, MOGAD, and infectious etiologies. Important additional considerations include primary CNS vasculitis, neurosarcoidosis, infection-related or post-infectious inflammatory CNS syndromes, lymphoproliferative disorders and other undefined autoinflammatory CNS conditions. The differential diagnoses were systematically assessed through clinical evaluation, neuroimaging and laboratory testing, with histopathology performed when indicated. In the second patient, a low anti-MOG titer was interpreted as potentially non-specific (13) and the persistently refractory disease course despite intensive immunotherapy made MOGAD less likely.

The pathogenesis of BS is thought to result from a dysregulated immune response to environmental triggers in genetically predisposed individuals. Enhanced activation of innate immune cells, particularly neutrophils and monocytes, leads to increased production of pro-inflammatory cytokines (14). CNS involvement is histopathologically characterised by perivascular inflammatory infiltrates composed mainly of lymphocytes and neutrophils, often accompanied by necrosis (7, 12). Probable NBS may present with prominent neutrophilic inflammation without clear vasculitis (15). The absence of detectable pathogens further supports a noninfectious, immune-mediated process (16). Collectively, these observations emphasize the diagnostic value of brain biopsies when standard diagnostic evaluations remain inconclusive.

Therapeutically, infliximab is considered one of the most effective treatments for NBS (17, 18). Infliximab was not used in these two case reports because, in case 1, the TNF-α inhibitor etanercept did not prevent CNS involvement of the disease, and in case 2 it was withheld due to concern for a central demyelinating disorder. This decision was based on the known risk of paradoxical disease exacerbation of demyelinating conditions such as multiple sclerosis and MOG-antibody-associated disease under TNF-α inhibitor therapy (19).

aHSCT represents a highly invasive immunotherapy with profound changes of the immune landscape and hereby partial or complete remission described in several cases of BS (11, 20). However, some reported cases describe a more ambiguous clinical response (9). Despite adequate mobilisation and conditioning according to EBMT guidance, both patients exhibited ongoing clinical inflammatory disease activity as well as radiological activity on MRI. It is conceivable that the inflammatory processes driving disease activity in these patients were not fully dependent on immune cell populations effectively eliminated by aHSCT. Persistence of tissue-resident immune cells, innate immune activation, or alternative autoinflammatory pathways may have contributed to ongoing disease activity despite immune reconstitution. Additionally, advanced or biologically distinct forms of Neuro-Behçet may not respond to immune reset strategies. Furthermore, post-transplant complications may have contributed to the clinical deterioration and complicate interpretation of the clinical outcome, especially in patient 1 (CMV reactivation, GI bleeding and lymphoma).

The atypical presentation of new cerebral MRI lesions in patient 2 is most probably attributable to the underlying disease. However, given their unusual morphology and distribution, a rare cerebral post-transplant lymphoproliferative disorder must also be considered in the differential diagnosis (21). Overall, NBS is a rare but devastating condition which should be considered in cases with atypical CNS involvement, even in the absence of established systemic Behçet criteria. Histopathological findings may be decisive and can guide therapeutic decisions that would otherwise be avoided in LETM such as in case 2. Nonetheless, management of patients with probable NBS remains challenging.

Several limitations should be acknowledged. First, both patients fulfilled criteria for probable rather than definite Neuro-Behçet syndrome, leaving open the possibility that the observed disease course was driven by a Neuro-Behçet-like inflammatory CNS disorder. Second, conclusions regarding treatment efficacy cannot be drawn from two individual cases. Third, both patients underwent aHSCT after prolonged disease duration and substantial neurological disability accumulation, potentially limiting the benefit of immune reconstitution therapy. Finally, detailed post-transplant immunological profiling was not available and therefore mechanistic explanations for treatment failure remain speculative. Furthermore, standardized outcome measure were limited to the EDSS, with primarily reflects motor function at higher scores and therefore does not adequately capture other clinically relevant domains such as cognition, upper extremity function, and quality of life.

In conclusion, these cases highlight the diagnostic and therapeutic challenges of severe Neuro-Behçet syndrome presenting with longitudinally extensive myelitis and brainstem involvement. Probable NBS should be actively considered as a differential diagnosis in patients with atypical inflammatory central nervous system presentations, even in the absence of established systemic criteria. Our observations further suggest that escalation therapies such as autologous hematopoietic stem cell transplantation may fail to control disease activity in aggressive cases, underscoring the need for careful patient selection and risk–benefit assessment. Finally, histopathological evaluation can provide decisive diagnostic information in complex cases where clinical and radiological findings remain inconclusive. Together, these findings emphasize the need for improved diagnostic strategies and more robust evidence to guide treatment decisions in this rare but severe condition.

## Data Availability

The original contributions presented in the study are included in the article/**Supplementary Material**. Further inquiries can be directed to the corresponding author.
