# Supplementary material for: Two cases of probable Neuro-Behçet syndrome treated with autologous HSCT
Source: Front Immunol. 2026 Jul 13;17:1864802. doi: 10.3389/fimmu.2026.1864802 (PMC13403935; doi:10.3389/fimmu.2026.1864802)
Supplement: Supplementary file 1 [file Supplementaryfile1.pdf]

Two cases of probable Neuro-Behçet Syndrome treated with autologous HSCT

Charlotte Schubert[1†], Lea I. Walter[2†], Marina Herwerth[2,3,4], Imke Metz[5], Jakob Nilsson[6], Ilijas Jelcic[2], Patrick Roth[2,3], Nicolaus Kröger[7], Ina Kötter[8], Christoph Heesen[1], Vivien Häußler[1*††], Veronika Kana[2††]

[1]Institute of Neuroimmunology and Multiple Sclerosis (INIMS) and Department of Neurology, University Medical Centre Hamburg-Eppendorf, Hamburg, Germany

[2]Department of Neurology, University Hospital Zurich and University of Zurich, Zurich, Switzerland

[3]Neuroscience Centre Zurich, University of Zurich and ETH Zurich, Zurich, Switzerland

[4]Institute of Pharmacology and Toxicology, University of Zurich, Zurich, Switzerland

[5]Institute of Neuropathology, University Medical Centre Göttingen, Göttingen 37075, Germany

[6]Neuroimmunology and Multiple Sclerosis Research Section, Department of Neurology, University of Zurich and University Hospital Zurich, Zurich, Switzerland

[7]Department of Stem Cell Transplantation, University Medical Centre Hamburg-Eppendorf, Hamburg, Germany

[8] Division of Rheumatology and Systemic Inflammatory Diseases, III. Department of Medicine, University Medical Centre Hamburg-Eppendorf, Hamburg, Germany.

†These authors contributed equally to this work and share first authorship

††These authors contributed equally to this work and share last authorship

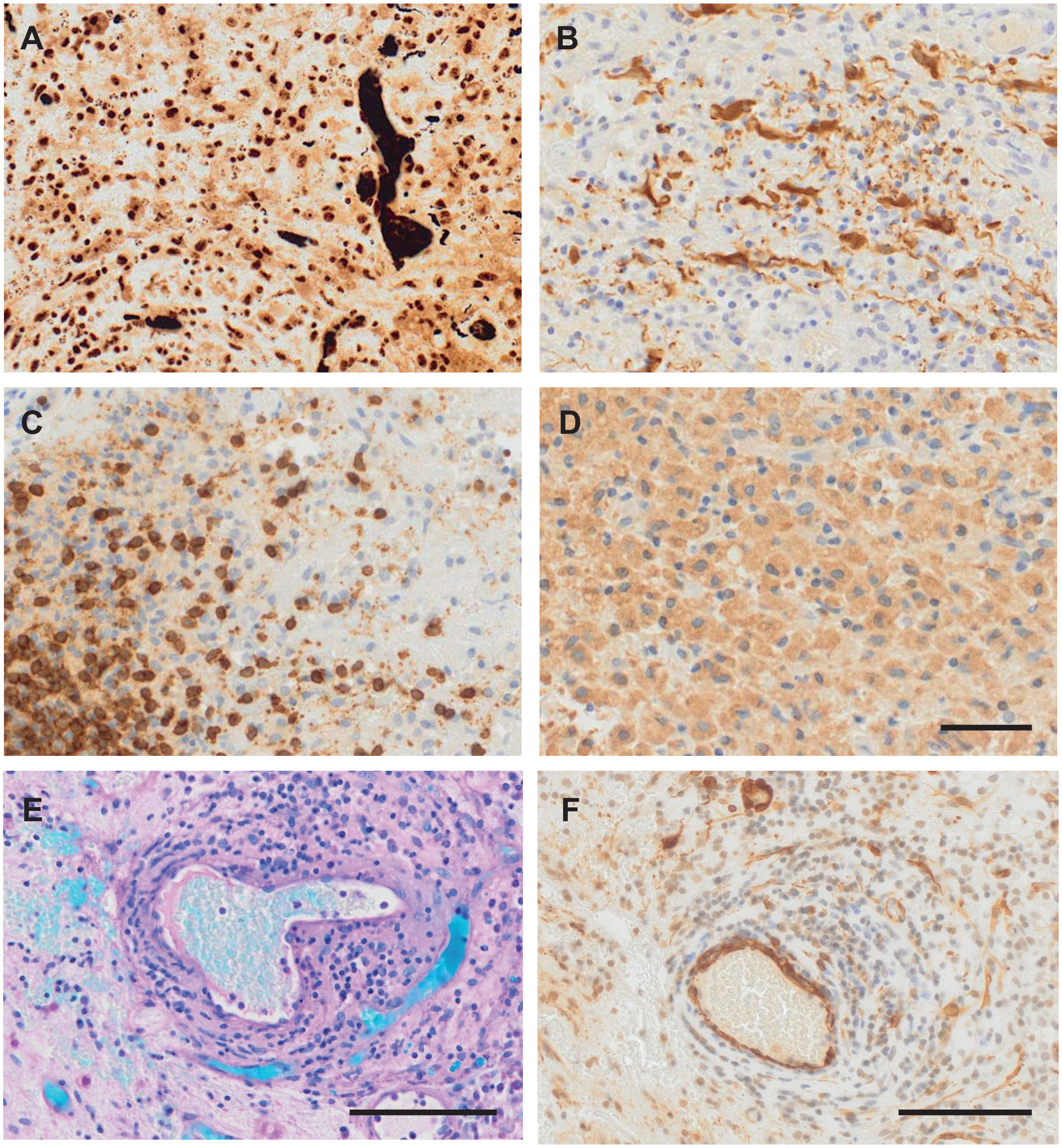

Supplementary Figure S1: Supporting histology shows an inflammatory necrotic lesion consistent with Behçet disease.

(A) Bielschowsky silver staining shows a complete loss of axons in the necrotic regions. **(B)** The necrotic lesions are also characterised by partially lost and damaged astrocytes (GFAP). The inflammatory infiltrate contains numerous lymphocytes, predominantly **(C)** CD8+ T lymphocytes, and **(D)** macrophages (KiM1P). Scale bar: 50 µm valid for (A) to (D). (E) Luxol fast blue/periodic acid–Schiff (LFB/PAS) staining demonstrates necrotizing inflammatory destruction of the brain parenchyma with loss of normal tissue architecture adjacent to the affected vessel. (F) Smooth muscle

actin (SMA) staining highlights disruption and splitting of the vessel wall architecture, indicating inflammatory involvement of the vessel wall in the absence of fibrinoid necrosis or thrombosis. Scale bar: 100 µm for (E) and (F).

Supplementary Table 1: Behçet disease manifestations according to the International Criteria for Behçet Disease (ICBD) and additional findings supporting the diagnosis of probable Neuro-Behçet syndrome.

**Clinical feature**	**ICBD points**	**Case 1**	**Case 2**
Oral aphthosis (recurrent oral ulcers)	2	Present	Absent
Genital aphthosis	2	Absent	Absent
Ocular involvement	2	Absent	Absent
Skin manifestations	1	Absent	Absent
Neurological manifestations	1	Present	Present
Vascular manifestations	1	Absent	Superficial thrombophlebitis
Positive pathergy test*	1	Negative	Not performed
**Supportive symptoms**			
HLA-B51 positivity	–	Present	Absent
Elevated inflammatory markers	–	Not reported	Intermittent CRP elevation
Histopathology compatible with Neuro-Behçet syndrome	–	Not available	Present
**Total ICBD score**		**3**	**2**

***optional**
